# The *Sclerotinia sclerotiorum*-inducible promoter *pBnGH17*^*D7*^ in *Brassica napus*: isolation, characterization, and application in host-induced gene silencing

**DOI:** 10.1093/jxb/erac328

**Published:** 2022-08-05

**Authors:** Li Lin, Jialin Fan, Panpan Li, Dongxiao Liu, Sichao Ren, Keyun Lin, Yujie Fang, Chen Lin, Youping Wang, Jian Wu

**Affiliations:** Key Laboratory of Plant Functional Genomics of the Ministry of Education, Yangzhou University, Yangzhou 225009, China; Key Laboratory of Plant Functional Genomics of the Ministry of Education, Yangzhou University, Yangzhou 225009, China; Key Laboratory of Plant Functional Genomics of the Ministry of Education, Yangzhou University, Yangzhou 225009, China; Key Laboratory of Plant Functional Genomics of the Ministry of Education, Yangzhou University, Yangzhou 225009, China; Key Laboratory of Plant Functional Genomics of the Ministry of Education, Yangzhou University, Yangzhou 225009, China; Jiangsu Key Laboratory of Crop Genomics and Molecular Breeding, Yangzhou University, Yangzhou 225009, China; Jiangsu Key Laboratory of Crop Genomics and Molecular Breeding, Yangzhou University, Yangzhou 225009, China; Key Laboratory of Plant Functional Genomics of the Ministry of Education, Yangzhou University, Yangzhou 225009, China; Key Laboratory of Plant Functional Genomics of the Ministry of Education, Yangzhou University, Yangzhou 225009, China; Jiangsu Key Laboratory of Crop Genomics and Molecular Breeding, Yangzhou University, Yangzhou 225009, China; Key Laboratory of Plant Functional Genomics of the Ministry of Education, Yangzhou University, Yangzhou 225009, China; Ghent University, Belgium

**Keywords:** *Brassica napus*, host-induced gene silencing, inducible promoter, *Sclerotinia sclerotiorum*, TGA7, TGACG motif

## Abstract

Sclerotinia stem rot (SSR), caused by *Sclerotinia sclerotiorum*, is among the most devastating diseases in *Brassica napus* worldwide. Conventional breeding for SSR resistance in *Brassica* species is challenging due to the limited availability of resistant germplasm. Therefore, genetic engineering is an attractive approach for developing SSR-resistant *Brassica* crops. Compared with the constitutive promoter, an *S. sclerotiorum*-inducible promoter would avoid ectopic expression of defense genes that may cause plant growth deficits. In this study, we generated a *S. sclerotiorum*-inducible promoter. *pBnGH17*^*D7*^, from the promoter of *B. napus* glycosyl hydrolase 17 gene (*pBnGH17*). Specifically, 5'-deletion and promoter activity analyses in transgenic *Arabidopsis thaliana* plants defined a 189 bp region of *pBnGH17* which was indispensable for *S. sclerotiorum*-induced response. Compared with *pBnGH17*, *pBnGH17*^*D7*^ showed a similar response upon *S. sclerotiorum* infection, but lower activity in plant tissues in the absence of *S. sclerotiorum* infection. Moreover, we revealed that the transcription factor BnTGA7 directly binds to the TGACG motif in *pBnGH17*^*D7*^ to activate *BnGH17*. Ultimately, *pBnGH17*^*D7*^ was exploited for engineering *Sclerotinia*-resistant *B. napus* via host-induced gene silencing. It induces high expression of siRNAs against the *S. sclerotiorum* pathogenic factor gene specifically during infection, leading to increased resistance.

## Teaser

We identified *pBnGH17*^*D7*^, a *Sclerotinia sclerotiorum-*inducible promoter, in *Brassica napus*. BnTGA7 binds the TGACG motif in *pBnGH17*^*D7*^to activate *BnGH17*during *S. sclerotiorum*infection*. pBnGH17*^*D7*^was applied to engineer *Sclerotinia*-resistant *B. napus*by HIGS.

## Introduction

Due to the world’s growing population and changing climate, powerful tools are needed to engineer desirable agronomic traits in crops that increase productivity. Compared with conventional breeding, genetic engineering enables the introduction, removal, or modification of desired genes in a specific crop with minimal modifications to the crop genome (Dong and Ronald, 2019). Three different types of promoters are typically employed to control transgene expression in plant genetic engineering: constitutive promoters, inducible promoters, and tissue-specific promoters. In plants, constitutive promoters such as the *Cauliflower mosaic virus* (CaMV) *35S* and maize *ubiquitin* promoters are most commonly used (Benfey and Chua, 1990; Cornejo *et al.*, 1993). However, constitutive promoters drive transgene expression throughout all stages of plant development, in most plant tissues, and under all conditions, making it challenging to control specific temporal and spatial expression of transgenes (Holtorf *et al.*, 1995; Sunilkumar *et al.*, 2002). In addition, constitutive promoters trigger a continuously high level of transgene expression, which often leads to unnecessary nutrition consumption and plant growth deficits (Hull *et al.*, 2000; Pino *et al.*, 2007). In contrast to constitutive promoters, transgenes driven by tissue-specific promoters could achieve optimal effectiveness (Koellhoffer *et al.*, 2015; Li *et al.*, 2019). Inducible promoters that regulate transgene expression in a desired temporal and/or spatial manner lessen the incidence of unexpected adverse effects on plant growth. Therefore, tissue-specific and inducible promoters are the preferred route in plant genetic engineering.

Plants deploy a wide range of immune defense strategies against pathogens that can be exploited to confer disease resistance through genetic engineering. However, genetic immunity to disease often comes with the cost of reduced plant growth and reproduction (Ning *et al.*, 2017; Guo *et al.*, 2018). Yield penalties caused by enhanced disease resistance have been described in several crop species. The wheat resistance (R) gene *Wsm1* conferred plant resistance to *Wheat streak mosaic virus*, but caused a relative 11–28% reduction in yield, even in unstressed conditions (Sharp *et al.*, 2002). A similar observation was made with powdery mildew-resistant barley, in which the *mlo* R gene-mediated resistance to *Bgh* (powdery mildew) reduced grain yield by 4% (Jørgensen, 1992). In *Arabidopis thaliana*, non-expresser of pathogenesis-related gene 1 (*AtNPR1*), a master immune regulatory gene, was a prime gene to be employed in disease resistance engineering, in that *AtNPR1* conferred resistance to diverse pathogens in different plant species (Cao *et al.*, 1998; Lin *et al.*, 2004; Quilis *et al.*, 2008; Wally *et al.*, 2009). However, constitutive overexpression of *AtNPR1* resulted in reduced plant height and yield loss in rice, limiting its potential application in broad-spectrum resistance (Quilis *et al.*, 2008). Thus, immune responses during genetic engineering should be precisely regulated to mitigate the cost of resistance. This can be achieved by inducing the expression of defense genes at particular times or in specific plant tissues (Karasov *et al.*, 2017). It is believed that expression of defense genes under the control of pathogen-inducible promoters could maintain the balance between plant growth and disease resistance during genetic engineering.

To date, a number of natural pathogen-inducible promoters have been isolated and identified in plants, including: the *Magnaporthe grisea*-inducible promoters *OsR2329*, *OsR2184*, and *OsPBZ1* in rice (Sasaki *et al.*, 2007); the *Phytophthora sojae*-inducible promoter *PcCMPG1* in *Petroselinum crispum* (Kirsch *et al.*, 2001); the *Bgh-* and *Rhynchosporium secalis*-inducible promoter *HvGER4c* in barley and wheat (Himmelbach *et al.*, 2010); the *Uncinula necator-*inducible promoter *VpSTS* in grapevine (Xu *et al.*, 2010); the *Xanthomonas axonopodis-*inducible promoter *NtpPPP1* in *Citrus sinensis* Osbeck (Zou *et al.*, 2014); and the *Erwinia amylovora-*inducible promoters *Ntstr246C*, *Ntsgd24*, and *StPgst1* in pear and apple (Malnoy *et al.*, 2003, 2006). However, research on pathogen*-*inducible promoters in *Brassica* crops is limited.

As a major oil crop worldwide, oilseed rape (*Brassica napus*) provides vegetable oil for humans and edible fodder for animals. However, the growth of oilseed rape is constantly threatened by Sclerotinia stem rot (SSR). This disease, caused by the broad-host-range fungal pathogen *Sclerotinia sclerotiorum*, leads to severe reduction in seed yield and quality worldwide. SSR resistance in *B. napus* is a quantitative trait, determined by multiple minor quantitative trait loci (QTLs) (Wu *et al.*, 2016a). However, none of the QTLs has been cloned, limiting their utilization in SSR resistance breeding. Reverse genetic analysis of SSR resistance has been conducted, and several defense genes have been identified (Ding *et al.*, 2021).

Genetic engineering for SSR resistance using the identified defense genes is a promising strategy for controlling SSR. However, overexpression of defense genes may cause plant growth deficits (Ning *et al.*, 2017). Therefore, the regulation of defense gene expression precisely controlled by *S. sclerotiorum-*inducible promoters is an optimal strategy to generate SSR-resistant varieties with stable *B. napus* yields. While two synthetic promoters containing pathogen-related *cis-*acting elements have been described that respond to *S. sclerotiorum* (Shokouhifar *et al.*, 2011a, b). To date, *S. sclerotiorum-*inducible promoters in plants have not been identified or explored.

Here, we identified an *S. sclerotiorum-*inducible promoter derived from *B. napus* and highlighted its potential application in agriculture. The crucial promoter region and the core *cis*-elements that respond to *S. sclerotiorum* were investigated by 5ʹ-deletion analysis and site-directed mutagenesis. The transcription factor (TF) that directly binds to this crucial promoter region was verified by yeast one-hybrid (Y1H) assay, dual-luciferase assay (dual-LUC), and EMSA. Finally, this promoter was used to engineer SSR-resistant *B. napus* and tested for its specificity. This research highlights the potential of a *S. sclerotiorum-*inducible promoter to facilitate precise genetic engineering of SSR-resistant *B. napus* and potentially other crops.

## Materials and methods

### Plant materials, abiotic stress and phytohormone treatments, and *S. sclerotiorum* inoculation

*Brassica napus* line J9712 was kindly provided by Professor Yongming Zhou (Huazhong Agricultural University, Wuhan, Hubei, China). Plants were grown in nutrient solution in the greenhouse for 4 weeks, and then the entire seeding plants were subjected to various treatments, including osmotic, cold, heat, and salt stresses, as well as to hydrogen peroxide (H_2_O_2_) and hormone treatments with salicylic acid (SA), abscisic acid (ABA), methyl jasmonate (MeJA), and ethephon (ETH), and *S. sclerotiorum* inoculation. For the osmotic stress and salt stress treatments, seedlings were transferred to nutrient solutions containing 15% polyethylene glycol 6000 (PEG6000) and 200 mM NaCl, respectively, and sampled at 6 h after treatment, as described by Li *et al*. (2021a, b). For the cold and heat treatments, seedlings were transferred to growth chambers with light intensity of ~300 µmol⋅m^−2^⋅s^−1^ at 4 °C and 42 °C, respectively, and sampled 1 h and 6 h post-treatment (Li *et al.*, 2021b). For the H_2_O_2_ and hormone treatments, seedlings were sprayed with 100 µM H_2_O_2_ (Mierek-Adamska *et al.*, 2019), 1 mM SA (Wang *et al.*, 2014a), 100 µM MeJA (Wang *et al.*, 2014a), 100 µM ABA (Li *et al.*, 2021b), and 100 µM ETH (Xue *et al.*, 2020), and sampled at 3 h and 6 h post-treatments. For *S. sclerotiorum* inoculation, the *S. sclerotiorum* isolate SS-1 was cultured on potato dextrose agar (PDA; Becton, Dickinson and Company, Franklin Lakes, NJ, USA), as described by Wu *et al.* (2013). Agar plugs of 7 mm in diameter with *S. sclerotiorum* were used for detached leaf inoculation of unfolded leaves of *B. napus* line J9712, as described by Wu *et al.* (2021). Mock-inoculated leaves were treated with 7 mm diameter agar plugs. Tissues extending 10 mm beyond the inoculation site on the leaves were harvested at 3, 6, and 12 h after *S. sclerotiorum* or mock inoculation and stored at –80 °C. Three biological replicates were performed, and five plants were used for each biological replicate.

### Promoter isolation and promoter::GUS vector construction

The 5ʹ-flanking region upstream of the translation start codon of *BnGH17* (BnaC01g21880D) was isolated from line J9712 using sequence-specific primers (Supplementary Table S1) that were based on reference genome sequences (Chalhoub *et al.*, 2014; Song *et al.*, 2020). The *cis*-regulatory elements of the *BnGH17* promoter (*pBnGH17*) were predicted using the online software PlantCARE (http://bioinformatics.psb.ugent.be/webtools/plantcare/html/) (Lescot *et al.*, 2002).

For promoter deletion analysis, three deletions, D1 (–500 to –1), D2 (–848 to –1), and D3 (–1260 to –1), were generated by PCR. Four other deletions, D4 (–1784 to –1261 and –500 to –1), D5 (–1430 to –1261 and –500 to –1), D6 (–1784 to –1607 and –500 to –1), and D7 (–1615 to –1427 and –500 to –1), were generated through splicing by the overlap extension PCR technique (Horton *et al.*, 2013) with four primers pairs (Supplementary Table S1). For site-directed mutagenesis analysis, splicing by the overlap extension PCR technique was used to introduce three mutations in the TGACG motifs of D7 at loci –1423/–1427. The primers pairs are listed in Supplementary Table S1.

To construct the promoter::GUS (β-glucuronidase) expression vector, promoter fragments were cloned into pBI101 at *Eco*RI and *Bam*HI restriction enzyme sites via homologous recombination (ClonExpress II One Step Cloning Kit, Vazyme, Nanjing, China).

### *A. thaliana* transformation and treatments

Promoter::GUS recombinant plasmids were introduced into *Agrobacterium tumefaciens* strain GV3101 by electroporation. *Arabidopsis thaliana* wild-type plants Columbia-0 (Col-0) were used for transformation via the floral dipping method (Zhang *et al.*, 2006). Seeds of the T_0_ generation were selected on Murashige and Skoog (MS) medium supplemented with kanamycin (50 mg l^–1^), and the positive plants were further verified by PCR. T_1_ and T_2_ transgenic plants were grown in nutrient soil and, after verification by PCR, they were transplanted for hormone treatments, *S. sclerotiorum* inoculation, and the GUS staining assay. All *A. thaliana* plants were grown in growth chambers under a 16 h light/8 h dark photoperiod (~300 µmol⋅m^−2^⋅s^−1^) at 22 °C during the day and 20 °C at night, and 60% relative humidity.

For *S. sclerotiorum* inoculation, 4-week-old unfolded leaves were detached from *A. thaliana* plants, and placed on agar for leaf inoculation. Mycelial agar plugs (2 mm in diameter) punched from the margin of a 2-day-old cultures of *S. sclerotiorum* grown on PDA were used as the inoculum and were closely appended to the adaxial surface of leaves, according to Wu *et al.* (2013). Mock-inoculated leaves were treated with 2 mm diameter agar plugs. The inoculated and mock-inoculated leaves were covered with plastic film to maintain moisture at 22 °C. At 12 h and 24 h post-inoculation (hpi), the inoculated leaves were collected for GUS staining and quantiative real-time PCR (qRT-PCR) analysis, respectively. For H_2_O_2_ and hormone treatments, 4-week-old *A. thaliana* plants were sprayed with 1 mM SA (Kovacs *et al.*, 2015), 200 µM MeJA (Jiang *et al.*, 2014), 7 mM ETH (Qiu *et al.*, 2015), and 100 μM H_2_O_2_ (Zhang *et al.*, 2003). At 6 h and 12 h post-treatment, *A. thaliana* leaves were collected for GUS staining and qRT-PCR analysis, respectively. GUS staining was performed on four independent transgenic lines for each treatment. For qRT-PCR analysis, three independent biological replicates were performed, each with three technical replicates.

### Histochemical GUS staining

Histochemical GUS staining was performed as described by Jefferson *et al.* (1987). Briefly, samples were incubated at 37 °C overnight in GUS staining buffer containing 0.5 mg ml^–1^ X-gluc, 0.5 mM potassium ferricyanide, 0.5 mM potassium ferrocyanide, 10 mM EDTA, 0.1% Triton X-100, and 10 mM sodium phosphate (pH 7.2). After that, the samples were bleached in 75% (v/v) ethanol and photographed using a stereomicroscope with a digital camera (EZ4W, Leica, Bensheim, Germany).

### Total RNA extraction and qRT-PCR analysis

Total RNA from different tissues and organs of *A. thaliana* and *B. napus* was extracted according to the TRIzol method using an RNAiso reagent kit (Vazyme) according to the manufacturer’s instructions. The total RNA was reverse transcribed into first-strand cDNA with a HiScript II 1st Strand cDNA Synthesis Kit (Vazyme). qRT-PCR was carried out using AceQ Universal SYBR qPCR Master Mix (Vazyme) in an ABI Step One Plus real-time PCR system (Applied Biosystems Inc., Foster City, CA, USA). The relative expression of each gene was calculated using the 2^-△△Ct^ method (Livak and Schmittgen, 2001). *BnUBC9* (BnaC08g12720D) and *BnUBC10* (BnaA10g06670D) in *B. napus* (Wu *et al.*, 2016b), *AtEF-1α* (At5g60390) and *AtUBQ10* (At5g53300) in *A. thaliana* (Yang *et al.*, 2020, Zhu *et al.*, 2013), and *SsActin* (SS1G_08733) and *Sstub1* (SS1G_04652) (Wu *et al.*, 2021) in *S. sclerotiorum* were used as reference genes. The qRT-PCR primers used are shown in Supplementary Table S1. All qRT-PCR experiments were performed with three biological replicates, each with three technical replicates.

### Y1H assay

A Y1H assay was performed as described by Ou *et al.* (2011). An *A. thaliana* TF library containing 1589 GAL4-AD-fused TFs was provided by Professor Lijia Qu (Peking University, Beijing, China). The *pBnGH17*^*D7*^ subfragment was amplified by PCR, cloned into a pHisi-1 vector, and then transformed into yeast strain YM4271 as bait. The *A. thaliana* TF pooled library strains and the bait clone were grown in SD-Leu and SD-His medium overnight, respectively. The pooled library strains and bait clones were mixed at equal volumes (20 μl per well) and transferred to new 2 ml 96-well plates with yeast extract peptone dextrose medium. After 1 d growth with shaking at 200 rpm at 30 °C, the mating products were diluted 10-fold with water and then the diluted mating products (10 μl per well) were plated on screening plates [SD-Leu -His+15 mM 3-aminotriazole (3-AT)] and subsequently grown for 3 d.

The point-to-point Y1H assay was carried out according to Yang *et al.* (2011). Briefly, the *pBnGH17*^*D7*^ subfragment was cloned into the pHIS2 vector as bait. The full-length coding sequences (CDSs) of the TGA TF genes *BnTGA7* (BnaA07g33790D) and *BnTGA3* (BnaC05g17700D) were amplified by PCR and cloned into the pGADT7 vector as prey. Two plasmids were co-transformed into yeast strain Y187. Transformed clones were cultured on SD/-His/-Leu/-Trp selective medium containing 50 mM 3-AT for 3 d at 30 °C. The p53HIS2 and pGAD-Rec2-53 vectors were used as positive controls, and the pHIS2 and pGAD-Rec2-53 vectors were used as negative controls.

### Dual-luciferase reporter gene assay

The full-length CDSs of *BnTGA7* and *BnTGA3* were cloned into the pGREEN II 62-SK vector to generate effector constructs. The *pBnGH17*^*D7*^ subfragment was inserted ahead of the firefly luciferase (LUC) gene in the pGREEN II 0800-LUC vector to generate a reporter construct. Then, recombinant effector and reporter constructs and the empty vector pGREEN II 62-SK were introduced into *A. tumefaciens* strain GV3101 (with the helper PSoup-P19 plasmid) by electroporation and used to infect *Nicotiana benthamiana* leaves in the light (16 h/day) at 25 °C for 2 d by *Agrobacterium*-mediated infiltration to induce transient gene expression (Hellens *et al.*, 2005). The activities of the firefly luciferase (LUC) and Renilla luciferase (REN) were determined with a Dual-Luciferase Reporter Kit (Vazyme) according to the manufacturer’s instructions and detected with a microplate reader (Tecan Spark, Tecan Trading AG, Zurich, Switzerland).

### EMSA

The full-length CDS of *BnTGA7* was cloned into the pGEX6P-1 vector to generate the glutathione *S*-transferase (GST)–BnTGA7 fusion protein, which was expressed in *Escherichia coli* strain BL21. Expression and purification of the GST–BnTGA7 protein were performed according to the manufacturer’s instructions (Transgen, Beijing, China). A 30 bp DNA sequence containing the TGACG sequence was synthesized by Beijing Qingke Biotechnology and labeled with an EMSA Probe Biotin Labeling Kit (Beyotime, Shanghai, China). EMSA was performed using a chemiluminescent EMSA kit according to the manufacturer’s instructions (Beyotime). In brief, GST–BnTGA7 and the labeled probe were incubated at 25 °C for 20 min in a reaction system containing EMSA/gel-shift binding buffer. For the cold competition, 200-fold unlabeled probe was added to the reaction mixtures. These reaction mixtures were loaded on an 8% native PAGE gel; after transferring to a nylon membrane, cross-linking, and blocking the proteins, the signals were detected with a chemiluminescent EMSA kit.

### Host-induced gene silencing (HIGS) of the *S. sclerotiorum* endo-polygalacturonase gene (*SsPG1*) driven by *pBnGH17*^*D7*^

*pBnGH17* ^*D7*^ was cloned into the intron-containing hairpin vector pMDC83-ihpRNAi at the *Pme*I and *Spe*I sites to replace the CaMV *35S* promoter via homologous recombination with the ClonExpress II One Step Cloning Kit (Vazyme). Then, the 381 bp CDS fragment (296–676 bp) of *SsPG1* was cloned into the vector in sense and antisense orientations (sense–intron–antisense cassette) via homologous recombination as described by Wu *et al.* (2021). The primers are shown in Supplementary Table S1.

The HIGS construct was transformed into *A. tumefaciens* strain GV3101 by electroporation and then transformed into *B. napus* line J9712 via *A. tumefaciens*-mediated hypocotyl transformation as described by Liu *et al.* (2021a). Positive transgenic *B. napus* plants were selected by PCR with specific primers (Supplementary Table S1). All transgenic *B. napus* plants used in this study were from the T_2_ generation.

The resistance of transgenic *B. napus* plants to *S. sclerotiorum* was assessed by detached leaf, cotyledon, and stem inoculation, according to Wu *et al.* (2021). Approximately 15 plants in each of the three replicates were assessed for each line. For cotyledon inoculation, both the J9712 and T_2_ transgenic lines were grown in growth chambers with light intensity of 300 µmol⋅m^−2^⋅s^−1^under a 16 h light/8 h dark photoperiod at 24 °C and 60% relative humidity. For detached leaf and stem inoculation, all plants were grown in the experimental field at Yangzhou University, Jiangsu, China. The field experiment was conducted using a randomized complete block design with three replications.

Small RNA sequencing was performed to determine the expression of target gene-specific siRNAs in HIGS transgenic plants. *Sclerotinia sclerotiorum*- and mock-inoculated leaves of two independent transgenic T_1_ lines (three plants for each treatment) were randomly selected and mixed for small RNA sequencing. Small RNA sequencing and analyses were performed as described by Wu *et al.* (2021).

### Determination of polygalacturonase (PG) activity

To quantify PG activity in *S. sclerotiorum*-inoculated leaves, tissues extending 10 mm beyond the inoculation site on leaves were harvested at 24 hpi and then ground into powder with liquid nitrogen. Up to 0.1 g of each sample was used to determine the enzyme activity with the Polygalacturonase assay Kit (Solarbio, Beijing, China), according to the manufacturer’s instructions. The absorbance was recorded at 540 nm using a Tecan Infinite microplate reader (Spark M200, Tecan Austria GmbH, Grodig, Austria). Enzyme activity was defined as the decomposition of polygalacturonic acid per g of sample per hour at 40 °C, pH 6.0 to produce 1 μmol of galacturonic acid.

### Statistical analysis

Significance analysis was performed with Student’s *t-*test for comparing two independent groups (*P<0.05 and **P<0.01) by SPSS 19.0 (IBM SPSS Statistics, New York, NY, USA).

## Results

### Screening of the *S. sclerotiorum*-inducible promoter in *B. napus*

**Fig. 1.**
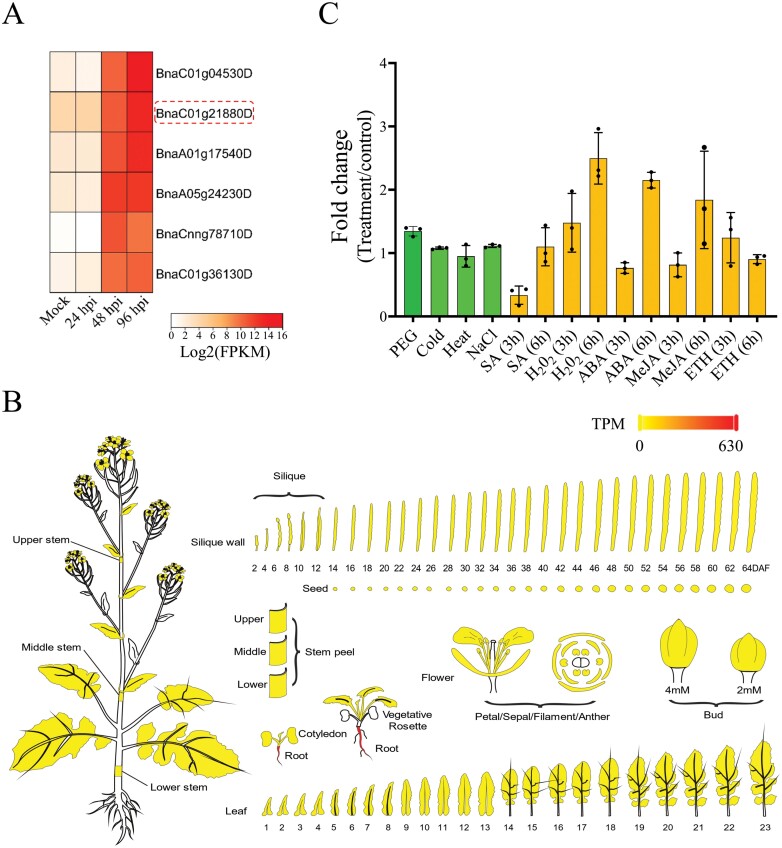
Expression patterns of *BnGH17* in *B. napus*. (A) The most strongly induced genes in *B. napus* after *S. sclerotiorum* infection, as determined by transcriptome sequencing (Wu *et al.*, 2016a). FPKM, fragments per kilobase of transcript per million fragments mapped; hpi, hours post-inoculation. (B) The eFP (electronic fluorescent pictograph) viewer displays the tissue expression patterns of *BnGH17* (BnaC01g21880D) based on the online transcriptome platform BnTIR (http://yanglab.hzau.edu.cn/BnTIR). Red indicates higher levels of transcript accumulation, and yellow indicates a lower level of transcript accumulation. TPM, transcripts per million mapped reads. (C) qRT-PCR analysis revealed the expression patterns of *BnGH17* under various stress conditions, including abiotic stress (PEG, cold, heat, and NaCl treatments; green bar); hormone treatments [salicylic acid (SA), abscisic acid (ABA), methyl jasmonate (MeJA), and ethephon (ETH)], and H_2_O_2_ treatment (orange bar). *BnUBC9* (BnaC08g12720D) was used as the reference gene. The values are presented as the mean ±SD of three independent biological replicates. Statistically significant differences between the control and each treatment were determined by Student’s *t*-test.

To determine the candidate genes induced by *S. sclerotiorum*, we performed transcriptomic analyses of *B. napus* before and after *S. sclerotiorum* infection using RNA sequencing (Wu *et al.*, 2016b). Among the differentially expressed genes between the *S. sclerotiorum*-challenged and mock-inoculated samples, the six most strongly induced genes, comprising one cysteine-rich secretory protein-, antigen 5-, and pathogenesis-related 1 protein (CAP)-encoding gene (BnaC01g04530D), two *BnGH17* genes (BnaC01g21880D and BnaA01g17540D), and three *legume lectin* (*BnLLP*) genes (BnaA05g24230D, BnaCnng78710D, and BnaC01g36130D) (Fig. 1A), were examined for their expression patterns in diverse tissues and under various stress conditions. Based on the transcriptomic database of diverse tissues of the *B. napus* cultivar ‘ZS11’ (http://yanglab.hzau.edu.cn/BnTIR) (Liu *et al.*, 2021b), BnaC01g04530D was eliminated from further analysis due to its ultrahigh expression level in roots [the transcripts per million mapped reads (TPM) value was 7558; Supplementary Fig. S1]. The three *BnLLP* genes were highly expressed in the root and silique walls and moderately expressed in the leaf (Supplementary Fig. S1), while the expression levels of the two *BnGH17* genes were extremely low in most of the tissues, except in roots (Fig. 1B; Supplementary Fig. S1). The expression patterns of *BnGH17* (BnaC01g21880D) under various stress conditions and with hormone treatments were determined, and *BnGH17* was not significantly induced by osmotic, cold, heat, or salt stress, or by H_2_O_2,_ SA, MeJA, ABA, or ETH treatment (Fig. 1C; Supplementary Fig. S2). Consequently, the *BnGH17* promoter was selected as the potential *S. sclerotiorum*-inducible promoter for further analysis.

### Isolation and characterization of the *BnGH17* promoter

To uncover the underlying molecular mechanism of the *BnGH17* promoter upon *S. sclerotiorum* infection, a 1784 bp promoter fragment upstream of the translational start site of *BnGH17* (BnaC01g21880D) was cloned. The known *cis*-regulatory elements in the promoter of *BnGH17* (*pBnGH17*) were analyzed with the PlantCARE online software (http://bioinformatics.psb.ugent.be/webtools/plantcare/html/) (Lescot *et al.*, 2002). In addition to core promoter elements (three putative TATA boxes and one putative CAAT box) (Kadonaga *et al.*, 1986; Ranish *et al.*, 1999), multiple other *cis*-elements were mapped out in *pBnGH17*, including four ABA-responsive elements (ABREs) (Guiltinan *et al.*, 1990), two MeJA-responsive elements (one CGTCA motif and one TGACG motif) (Wang *et al.*, 2011), two WRKY-binding sites (W boxes) (Rushton *et al.*, 1995), one basic helix–loop–helix (bHLH)-binding site (CANNTG motif) (Blackwell and Weintraub, 1990), one ethylene-responsive element (GCC box) (Ohme-Takagi and Shinshi, 1995), and four pathogen- and salt-inducible elements (GT-1 elements) (Park *et al.*, 2004) (Table 1). Our results indicated that *pBnGH17* is probably a stress-responsive promoter.

**Table 1.** Putative known *cis*-acting elements in the *BnGH17* promoter sequence

*Cis*-element	Description	Motif position^*a*^	Reference
ABRE	ABA-responsive element	–1430, –1367, –1246, –517	Guiltinan *et al.* (1990)
CGTCA motif	MeJA-responsive element	–493	Wang *et al.* (2011)
TGACG motif	MeJA-responsive element	–1432	Wang *et al.* (2011)
W-box	WRKY-binding site	–835, –237	Rushton *et al.* (1995)
CANNTG motif	bHLH-binding site	–1688	Blackwell *et al.* (1990)
GCC box	Ethylene-responsive element	–1219	Ohme-Takagi *et al.* (1995)
GT-1	Salt- and pathogen-responsive element	–862, –548, –409,	Park *et al.* (2004)
TATA box	Core promoter element	–966, –799, –131	Ranish *et al.* (1999)
CAAT box	Core promoter element	–61	Kadonaga *et al.* (1986)

^*a*^ Motif positions are indicated relative to the start codon ATG.

**Fig. 2.**
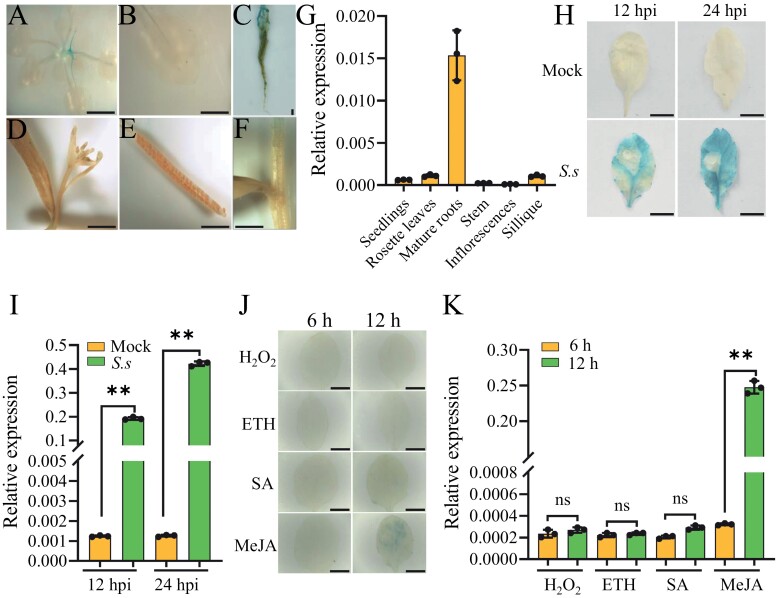
GUS expression analysis in T_2_ transgenic *A. thaliana* plants harboring the *pBnGH17*:*GUS* fusion. (A–G) Histochemical GUS staining (A–F) and qRT-PCR analysis of *GUS* expression (G) in diverse tissues, namely 10-day-old seedings (A), rosette leaves (B), mature roots (C), inflorescences (D), siliques with developing seeds (E), and stems (F). (H–K) Histochemical GUS staining (H, J) and qRT-PCR analysis of *GUS* expression (I, K) in leaves after *S. sclerotiorum* inoculation (H. I), as well as H_2_O_2_, salicylic acid (SA), methyl jasmonate (MeJA), and ethephon (ETH) treatments (J, K). The scale bars in (H) and (J) represent 5 mm. For qRT-PCR analysis, *AtEF-1α* (At5g60390) was used as the reference gene. The values are presented as the mean ±SD of three independent biological replicates. The asterisks indicate significant differences (**P<0.01, Student’s *t*-test). hpi, hours post-inoculation. *S.s*, *S. sclerotiorum*. Scale bars=5 mm.

To test its activity *in vivo*, *pBnGH17* was fused with *GUS* and transformed into wild-type *A. thaliana* plants. Histochemical GUS staining was carried out in different tissues of *A. thaliana*, including 10-day-old seedlings, rosette leaves, mature roots, inflorescences, siliques with developing seeds, and stems. GUS activity was slightly detected in seedlings and mature roots, but not in other tissues (Fig. 2A–F) during different growth stages. Relative expression of the *GUS* gene, determined by qRT-PCR, was consistent with the observed GUS staining pattern (Fig. 2G; Supplementary Fig. S3A).

In addition, the GUS staining pattern was visible in *pBnGH17::GUS* transgenic *A. thaliana* leaves treated with *S. sclerotiorum* at 12 hpi, and was much stronger at 24 hpi (Fig. 2H). In contrast, GUS activity was not detectable in the leaves of the mock-inoculated controls (Fig. 2H). In this case, the relative expression of *GUS* detected by qRT-PCR was also consistent with the GUS staining results: *GUS* expression increased by nearly 200-fold at 12 hpi, and then by 400-fold at 24 hpi as compared with the mock-inoculated controls (Fig. 2I; Supplementary Fig. S3B).

The qRT-PCR results showed that the expression of *BnGH17* was not induced by H_2_O_2_, SA, MeJA, or ETH treatments (Fig. 1C), even though several hormone-responsive elements were identified in the promoter region (Table 1). To further confirm this result, we examined GUS activity in *pBnGH17::GUS* transgenic *A. thaliana* after H_2_O_2_, SA, MeJA, and ETH treatments. Unsurprisingly, GUS staining was not detected at 6 h and 12 h after the H_2_O_2_, SA, and ETH treatments. While it was not detected 6 h after the MeJA treatment, weak GUS staining was detected 12 h later (Fig. 2J), consistent with *GUS* expression determined by qRT-PCR (Fig. 2K; Supplementary Fig. S3C). Collectively, our analysis supported the view that *pBnGH17* is a *S. sclerotiorum*-inducible promoter containing an *S. sclerotiorum*-inducible fragment.

### Deletion analysis of *pBnGH17* in transgenic *A. thaliana*

**Fig. 3.**
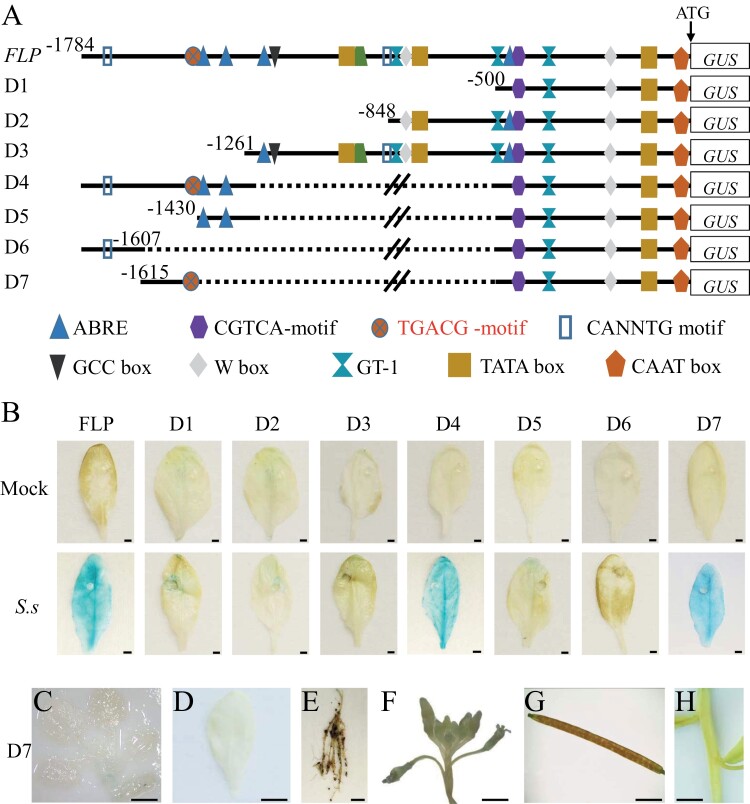
Deletion analysis of *pBnGH17* in transgenic *A. thaliana* (T_2_). (A) Schematic map of 5ʹ deletions of *pBnGH17*. FLP, full-length promoter. D1–D7, seven promoter deletions. Putative *cis*-acting elements in *pBnGH17* are represented by different shapes and colors. All positions are indicated relative to the start codon ATG. (B) Histochemical GUS staining of transgenic *A. thaliana* harboring the *pBnGH17* deletion*::GUS* fusions 24 h post-inoculation with *S. sclerotiorum* (*S.s*). (C–H) Histochemical GUS staining of the *pBnGH17*^*D7*^*::GUS* construct in diverse tissues under normal growth conditions: (C) 10-day-old seedings, (D) rosette leaves, (E) mature roots, (F) inflorescences, (G) siliques with developing seeds, and (H) stems. Scale bars=5 mm.

To define the *cis*-regulatory sequences enabling response to *S. sclerotiorum* infection, a series of 5ʹ deletions of *pBnGH17* were generated (Fig. 3A). Each of these fragments was further fused with *GUS* and transformed into *A. thaliana*. Histochemical GUS staining was performed with the leaves of transgenic *A. thaliana* inoculated with and/or without *S. sclerotiorum*. Initially, we generated three sequences, D1 (–500 to –1), D2 (–848 to –1), and D3 (–1260 to –1). Unexpectedly, GUS activity was not detectable in the transgenic *A. thaliana* plants after *S. sclerotiorum* inoculation (Fig. 3B), indicating that the fragment in the –1784 to –1261 region contains a compulsory molecular component for triggering GUS expression after *S. sclerotiorum* infection.

To further narrow down the region of *pBnGH17* that is responsible for the *S. sclerotiorum* infection, we generated another four deletions and linked them to putative core promoter regions, named D4–D7 (Fig. 3A). Interestingly, the D4 and D7 constructs in transgenic *A. thaliana* exhibited GUS activity after *S. sclerotiorum* infection as strong as the full-length *pBnGH17* construct in transgenic *A. thaliana* (Fig. 3B). However, the GUS staining pattern did not develop in the plants harboring D5 and D6 after *S. sclerotiorum* infection (Fig. 3B), indicating that the 189 bp promoter region located between positions –1615 and –1427 in *pBnGH17* is fundamentally essential for *S. sclerotiorum* infection responsiveness.

We next compared the GUS activity pattern between full-length *pBnGH17* and D7 in different plant tissues. Consistent with plants harboring full-length *pBnGH17*, GUS activity of the D7 construct was undetectable in the rosette leaves (Fig. 3D), inflorescences (Fig. 3F), siliques with developing seeds (Fig. 3G), and stems (Fig. 3H) of transgenic *A. thaliana* under normal growth conditions. In addition, the D7 construct also abolished GUS activity that was shown in seedlings and mature roots in full-length *pBnGH17* transgenic plants at the growth stage without stress (Fig. 3C, E). Hence, *pBnGH17*^*D7*^ was assumed to be an ideal inducible promoter to drive transgene expression during *S. sclerotiorum* infection.

### BnTGA7 interacts with the TGACG motif in *pBnGH17*

**Fig. 4.**
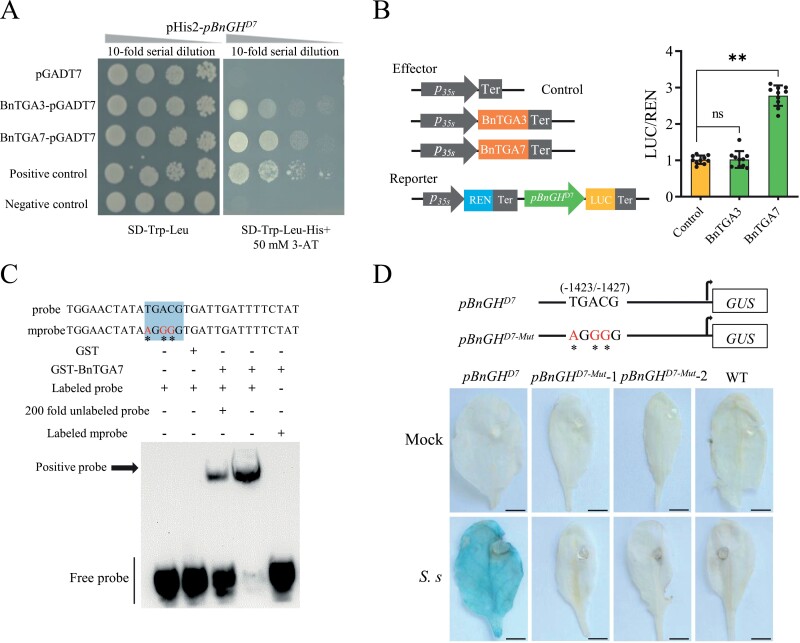
BnTGA7 directly binds to and transactivates *pBnGH17*. (A) Yeast one-hybrid assay of the binding activity of BnTGA3/BnTGA7 with *pBnGH17*^*D7*^. Positive controls, p53HIS2 and pGAD-Rec2-53. Negative controls, pHIS2 and pGAD-Rec2-53. (B) Dual-luciferase reporter assay of the interaction between BnTGA3/BnTGA7 and *pBnGH17*^*D7*^ in *N. benthamiana* leaves. The LUC/REN value of the control was set as 1 for calibration. The error bars indicate the SD. Statistical significance was determined by Student’s *t*-test (**P<0.01). (C) EMSA of the specific binding of recombinant BnTGA7 protein to the TGACG motif of *pBnGH17*^*D7*^. Underlining signifies the TGACG motif sequence, and asterisks represent the mutated base in the TGACG motif. GST, GST–BnTGA7, labeled probe, labeled mutant probe, and 200-fold unlabeled probe were present (+) or absent (–) in each reaction. (D) Histochemical GUS staining of two independent transgenic *A. thaliana* lines (T_1_) harboring the mutated promoter fragment *pBnGH17*^*D7-Mut*^::*GUS* fusion at 24 h post-inoculation with *S. sclerotiorum* (*S.s*). In *pBnGH17*^*D7-Mut*^, the TGACG motif was mutated to AGGGG. Col-0 (WT) was the negative control. Transgenic plants harboring the *pBnGH17*^*D7*^::*GUS* fusion were the positive control.

To identify the putative transcription regulators that interact with *pBnGH17* to mediate *S. sclerotiorum*-inducible gene expression, we screened a library of 1589 GAL4-fused *A. thaliana* TFs in a mating-based Y1H assay with the –1615 to –1427 region of *pBnGH17* (*pBnGH17*^–1615 to –1427^) as bait (Ou *et al.*, 2011). At1g77920 (AtTGA7) and At1g22070 (AtTGA3) were isolated in this high-throughput *A. thaliana* TF screening system. We then cloned the full-length CDS of *BnTGA7* and *BnTGA3* in *B. napus.* Using a point-to-point Y1H assay, we confirmed that both BnTGA3 (BnaC05g17700D) and BnTGA7 (BnaA07g33790D) interacted with *pBnGH17*^–1615 to –1427^ (Fig. 4A).

We further employed a dual-LUC reporter system in *N. benthamiana* to examine the transcriptional activity of BnTGA3 and BnTGA7 *in vivo*. The *pBnGH17*^*D7*^ promoter-driven firefly luciferase (LUC) reporter and 35S promoter-driven Renilla luciferase (REN, the internal control) were co-introduced into the plasmid as the reporter. Plasmids with or without *BnTGA3*/*BnTGA7* were used as the effector. The LUC:REN ratio, reflecting the transcriptional activity of the *pBnGH17*^*D7*^ promoter, was monitored after both the effector and reporter were transiently co-expressed in *N. benthamiana*. The co-expression of *pBnGH17*^*D7*^*::LUC*/*p35S::REN* and BnTGA7**,** but not BnTGA3, remarkably increased the LUC:REN ratio (Fig. 4B), suggesting that BnTGA7 activated *pBnGH17*^*D7*^ promoter-driven transcription *in vivo*.

TGA7, belonging to the TGA TF family, regulated the expression of defense genes (such as *pathogenesis-related 1*, *PR-1*) by directly binding to the TGACG motif in *A. thaliana* (Shearer *et al.*, 2009). As one putative TGACG motif was identified within *pBnGH17*^–1615 to –1427^, we subsequently tested if BnTGA7 directly binds to the TGACG motif of *pBnGH17* through EMSA *in vitro*. The GST–BnTGA7 recombinant protein was capable of binding to probes containing the TGACG motif, whereas the GST protein alone was not functional (Fig. 4C). The intensity of the binding signals decreased after the addition of unlabeled wild-type competitors (Fig. 4C). When the labeled probe was replaced with a mutated probe, the binding was completely abolished (Fig. 4C). Moreover, the change in expression of *BnTGA7* in the leaves of J9712 was detected after *S. sclerotiorum* inoculation. The transcript level of *BnTGA7* was slightly induced at 3 hpi, reached a peak at 6 hpi, and finally recovered at 12 hpi (Supplementary Fig. S4A, B). The expression of *BnGH17* was slightly induced at 3 hpi and kept increasing until 12 hpi (Supplementary Fig. S4C, D), suggesting that *BnTGA7* functions upstream of *BnGH17* and activates the expression of *BnGH17* in response to *S. sclerotiorum*.

To further study the contribution of the identified *cis*-element, the TGACG motif, on *S. sclerotiorum*-inducible gene expression, the TGACG motif of *pBnGH17*^*D7*^ was mutated to AGGGG, and the mutated promoter fragment *pBnGH17*^*D7-Mut*^ was fused with the *GUS* gene (Fig. 4D). GUS activities were completely abolished in transgenic *A. thaliana* lines carrying the *pBnGH17*^*D7-Mut*^*::GUS* fusion after *S. sclerotiorum* infection (Fig. 4D), further supporting our conclusion that the TGACG motif is the core motif for *S. sclerotiorum* response (Fig. 4D).

Taken together, the results of the Y1H assay, dual-LUC assay, EMSA, and site-directed mutagenesis of the TF-binding site suggested that BnTGA7 directly binds to the TGACG motif of *pBnGH17* and activates *pBnGH17-*driven transcription after *S. sclerotiorum* infection.

### Application of *pBnGH17*^*D7*^ to engineer *Sclerotinia*-resistant *B. napus*

**Fig. 5.**
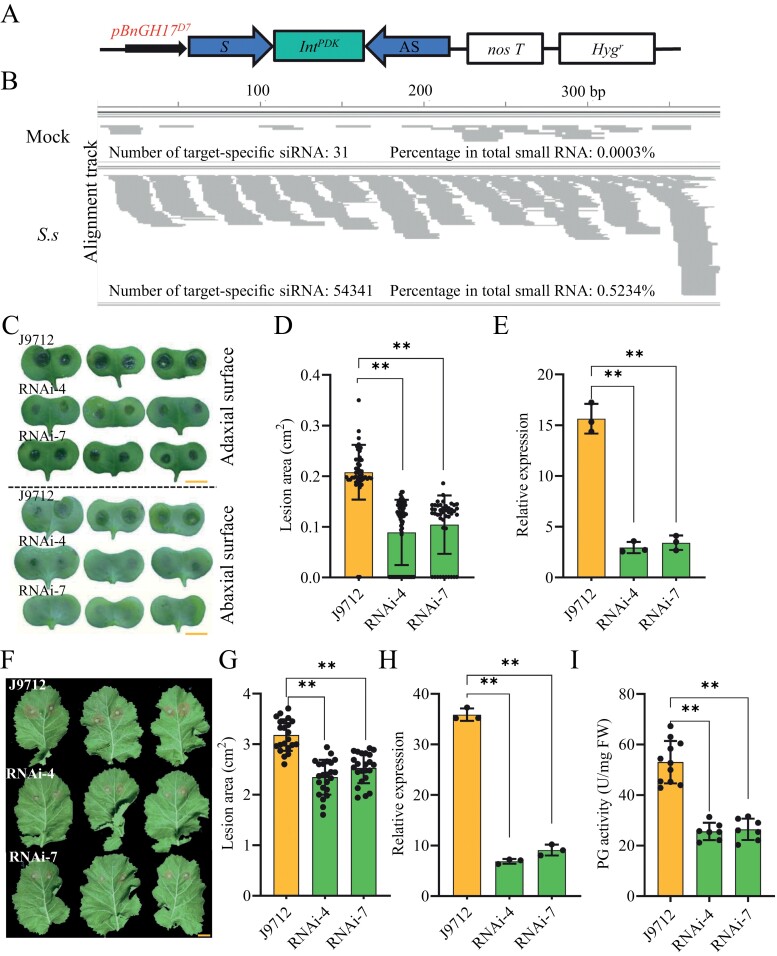
Host-induced gene silencing (HIGS) of the *S. sclerotiorum* endo-polygalacturonase gene (*SsPG1*) in *B. napus*. (A) Schematic of the HIGS construct. The RNAi expression cassette was driven by *pBnGH17*^*D7*^. Sense (S) and antisense (AS) fragments were inserted between *pBnGH*^*D7*^ and the nos terminator (nos T), forming a sense–*pyruvate orthophosphate dikinase* (*PDK*) intron (Int^*PDK*^)–antisense cassette. *Hyg*^*r*^, hygromycin phosphotransferase selection marker gene. (B) Small RNA profiling of *SsPG1* in uninoculated leaves (mock) and inoculated leaves (*S.s*) of HIGS transgenic T_1_ plants. The alignment tracks were obtained by mapping the Illumina sequence reads to the 381 bp sequence of *SsPG1*. (C–D, F–G) Assessment of disease resistance in HIGS transgenic *B. napus* to *S. sclerotiorum* as determined by cotyledon inoculation (C, D) and detached leaf inoculation (F, G). Disease lesions were photographed and measured 48 h post-inoculation (hpi). Scale bars=2 cm. (E, H) The expression levels of *SsPG1* in *S. sclerotiorum*-infected *B. napus* cotyledons (E) and leaves (H). Values were normalized to the fungal endogenous control gene *Sstub1* (SS1G_04652). The values are presented as the mean ±SD of three independent biological replicates. The asterisks indicate significant differences (**P<0.01, Student’s *t*-test). (I) The activity of polygalacturonase (PG) was determined in *S. sclerotiorum*-infected *B. napus* leaves at 24 hpi. Error bars indicate the SDs. Statistical significance was determined by Student’s *t-*test (**P<0.01).

PG is one of the most crucial cell wall-degrading enzymes in *S. sclerotiorum* pathogenicity (Amselem *et al.*, 2011). HIGS has been shown to induce gene silencing in pathogens by *in planta* expression of dsRNA or hairpin RNAs (hpRNAs) homologous to essential and/or pathogenicity genes of pathogens, conferring engineered plant protection from infection (Nowara *et al.*, 2010). It was recently revealed that HIGS of a pathogenic factor gene (endo-polygalacturonase gene, *SsPG1*) of *S. sclerotiorum* could be an effective strategy for controlling Sclerotinia rot in *B. napus* (Wu *et al.*, 2021). Therefore, we further investigated whether *pBnGH17*^*D7*^ is able to induce the expression of siRNAs in HIGS transgenic plants exclusively upon *S. sclerotiorum* infection. The HIGS target sequence of *SsPG1* was inserted into the intron-containing hairpin vector pMDC83-ihpRNAi to generate the HIGS construct, which is composed of the hygromycin phosphotransferase selection marker gene, the *pBnGH17*^*D7*^ promoter, a spacer sequence (PDK intron), and the nopaline synthase terminator (Fig. 5A).

The HIGS construct was transformed into the *B. napus* line J9712, and five independent T_0_-positive transgenic plants were obtained. To determine the expression of complementary siRNAs in HIGS transgenic plants, leaves of two transgenic T_1_ lines (RNAi-4 and RNAi-7) were harvested 24 h after *S. sclerotiorum* or mock inoculation and mixed for small RNA sequencing. In the mock inoculation sample, only 31 siRNAs derived from the HIGS construct were detected, accounting for 0.0003% of all small RNAs detected in this library (Fig. 5B). However, after inoculation with *S. sclerotiorum*, the abundance of the specific siRNAs (54 341) in the HIGS transgenic plants was vastly elevated, accounting for 0.5234% of the total small RNAs detected in this library (Fig. 5B). The most abundant siRNAs were 21–24 nt in length (Supplementary Fig. S5) and were distributed across the target gene region (Fig. 5B). Thus, our data supported that *pBnGH17*^*D7*^ triggered high expression of specific siRNAs in HIGS transgenic plants only during *S. sclerotiorum* infection.

Next, we evaluated the SSR resistance of the HIGS transgenic lines (T_2_) with cotyledon inoculation. Noticeable water-soaked lesions initially appeared on the adaxial surface of the cotyledons of J9712 at ~24 hpi and quickly extended to the abaxial surface; symptoms become even more severe after 48 hpi (Fig. 5C). In contrast, the fungus-induced water-soaked lesions appeared only on the adaxial surface at 48 hpi in most of the cotyledons of HIGS transgenic lines (Fig. 5C). In addition, 31.1% and 21.7% of the cotyledons of transgenic lines RNAi-4 and RNAi-7 were not successfully infected by *S. sclerotiorum*. The average lesion area on cotyledons of the transgenic lines was reduced by 51.8–58.2% compared with those of J9712 (Fig. 5D). The relative expression levels of *SsPG1* on the *S. sclerotiorum*-inoculated cotyledons of RNAi-4 and RNAi-7 were reduced by 81.2% and 78.1%, respectively, at 48 hpi compared with that in J9712 (Fig. 5E; Supplementary Fig. S6A), indicative of an enhanced resistance of these HIGS transgenic lines to *S. sclerotiorum* caused by target gene silencing.

To investigate whether the *S. sclerotiorum-*resistant phenotype occurs in other plant tissues, we repeated the experiments in detached leaves and stems. At 48 hpi, the lesion area on leaves of transgenic lines RNAi-4 and RNAi-7 was reduced by 26.2% and 20.1%, respectively, compared with those on the J9712 leaves at 48 hpi (Fig. 5F, G). At 7 dpi, the lesion lengths on stems of transgenic lines RNAi-4 and RNAi-7 were reduced by 25.2% and 23.1%, respectively, compared with those on the J9712 stems at 7 dpi (Supplementary Fig. S7A, B). The relative expression levels of *SsPG1* in transgenic lines RNAi-4 and RNAi-7 were reduced by 74.6% and 80.8%, respectively, at 24 hpi compared with that in J9712 (Fig. 5H; Supplementary Fig. S6B). Furthermore, the decreased expression of *SsPG1* in *S. sclerotiorum* at 24 hpi resulted in lower PG activity on the leaves of HIGS transgenic plants during infection than in the J9712 plants (Fig. 5I).

We critically compared the agronomic traits between the HIGS transgenic and wild-type plants. Our data indicated that the crop yield and the quality of the HIGS transgenic plants was not significantly influenced (Supplementary Table S2). It is hence conceivable that transgene expression driven by *pBnGH17*^*D7*^ is induced after *S. sclerotiorum* infection, thereby preventing unnecessary negative impacts on plant growth and development.

## Discussion

### *pBnGH17* ^*D7*^ activity is highly induced by *S. sclerotiorum*

In the past two decades, QTL mapping and genome-wide association studies have uncovered the genetic architecture of SSR resistance in oilseed rape (Ding *et al.*, 2021), and a considerable number of SSR resistance QTLs have been identified. However, none of them has been subjected to fine-mapping or map-based cloning, which may be attributable to the difficulty of identifying resistance phenotypes of complex plant–microbe–environment interactions. This dilemma has limited the utilization of resistance QTLs in SSR resistance breeding. Thus, genetic engineering for resistance to *S. sclerotiorum* is a promising strategy for controlling SSR. To this end, the *S. sclerotiorum-*inducible promoter is valuable for driving defense gene expression in response to *S. sclerotiorum* infection with high specificity.

To date, tissue-specific promoters and abiotic stress-inducible promoters have been identified in *B. napus*, including the seed-specific promoter *Napin* (Sohrabi *et al.*, 2015), the anther-specific promoter *Sta 44* (Hong *et al.*, 1997), the flower-specific promoters *FSP046* and *FSP061* (Li *et al.*, 2019), and the cold-inducible promoter *BN115* (Sangwan *et al.*, 2001). However, to the best of our knowledge, *S. sclerotiorum*-inducible promoters in *Brassica* have not been reported.

Collectively, our data revealed that *pBnGH17*, especially *pBnGH17*^*D7*^, is an *S. sclerotiorum*-inducible promoter. *pBnGH17* and *pBnGH17*^*D7*^ activity are induced by *S. sclerotiorum*. While *BnGH17* was expressed at a low level in most plant tissues (only root-specific expression) under normal conditions, expression was highly induced upon *S. sclerotiorum* infection, but not by other abiotic stresses or hormone treatments (Fig. 1; Supplementary Fig. S2). In addition, we confirmed that the activity of the *BnGH17* promoter in transgenic *A. thaliana* was consistent with the *BnGH17* expression pattern in *B. napus* (Fig. 2; Supplementary Fig. S3). Although the activity of full-length *pBnGH17* was observed in the roots of transgenic *A. thaliana* (Fig. 2A, C), the promoter deletion *pBnGH17*^*D7*^ almost completely abolished this activity (Fig. 3C, E). These data suggested that *pBnGH17*, especially *pBnGH17*^*D7*^, is potentially useful for SSR resistance breeding

### The potential application of *pBnGH17*^*D7*^ for genetic engineering of resistance to *S. sclerotiorum*

In the past few decades, the molecular mechanism of resistance to SSR in *B. napus* has been systematically investigated. To date, several *S. sclerotiorum* resistance genes have been identified through functional genomic analysis, including *BnMPK3* (Wang *et al.*, 2019); *BnMPK4* (Wang *et al.*, 2009); *BnMPK6* (Wang *et al.*, 2020b); *BnWRKY33* (Wang *et al.*, 2014b; Liu *et al.*, 2018); *BnWRKY15* (Liu *et al.*, 2018); *BnWRKY70* (Sun *et al.*, 2018); *BnMKK4*, *BnWRKY28*, and *BnVQ12* (Zhang *et al.*, 2021, Preprint); *BnNPR1* (Wang *et al.*, 2020a); *BnMED16* (Hu *et al.*, 2021); and *BnCCR2* (Liu *et al.*, 2021a). Overexpression or knockout these defense-related genes might incredibly enhance resistance to *S. sclerotiorum* in *B. napu*s (Liu *et al.*, 2018; X. Wang *et al.*, 2020; Wang *et al.*, 2020a; Zhang *et al.*, 2021, Preprint); however, DNA mutations and alterations in the expression of these defense-related genes often negatively influenced plant growth and yield (Ning *et al.*, 2017). Unfortunately, yield penalties caused by enhanced SSR resistance, to date have not received much consideration, despite some evidence provided in studies on *A. thaliana*, rice and *N. benthamiana*. To name a few, overexpression of constitutively active *BnMKK4*^*DD*^ induced hypersensitive cell death in *N. benthamiana* leaves (Zhang *et al.*, 2021, Preprint); constitutive expression of active *AtMPK3* resulted in a dwarf phenotype in *A. thaliana* (Genot *et al.*, 2017); and overexpression of the *A. thaliana* gene *AtNPR1* in rice led to height reduction and yield loss (Quilis *et al.*, 2008). Therefore, strictly controlling defense-related gene expression is gaining more and more attention. In this study, our data demonstrated that *pBnGH17*^*D7*^ is useful to precisely regulate the expression of defense-related genes in a *S. sclerotiorum*-inducible manner, while not incurring the negative effects inherent to the use of constitutive promoters.

Compared with *pBnGH17*, *pBnGH17*^*D7*^ is a desirable *S. sclerotiorum*-inducible promoter for the genetic engineering of SSR-resistant crops. The activities of *pBnGH17* were at low levels in the majority of plant tissues, but were relatively high in roots (Fig 2A, C). However, *pBnGH17*^*D7*^ exhibited very low activity in all tissues under normal conditions (Fig. 3C–H), and thus may have minimal adverse impacts on *B. napus* growth and development. When *pBnGH17*^*D7*^ was employed to engineer HIGS-based *Sclerotinia*-resistant *B. napus*, *pBnGH17*^*D7*^-triggered expression of high levels of specific siRNAs only occured after *S. sclerotiorum* infection (Fig. 5B; Supplementary Fig. S5), thereby preventing siRNA expression in all tissues and throughout all stages of plant development. These results demonstrated the potential utility of *pBnGH17*^*D7*^ for minimizing the yield penalty associated with enhanced SSR resistance in *B. napus.*

### BnTGA7 regulates the expression of *BnGH17* in response to *S. sclerotiorum* infection

In this study, we identified TFs that can interact with *pBnGH17*^*D7*^ with the high-throughput Arabidopsis TF screening system (Ou *et al.*, 2011). We clarified that BnTGA7 directly binds to the TGACG motif of *pBnGH17* and activates the expression of *BnGH17* (Fig. 4). Moreover, *BnTGA7* was significantly up-regulated after *S. sclerotiorum* infection (Supplementary Fig. S4A, B), suggesting the potential role of *BnTGA7* in SSR resistance. TGA family members have been implicated as important plant defense regulators possibly through physical interaction with the known master immune regulator NPR1 (Kesarwani *et al.*, 2007). To date, seven of 10 *A. thaliana* TGA TFs (TGA1–TGA7) have demonstrated interplay with NPR1 (Després *et al.*, 2000; Kesarwani *et al.*, 2007; Shearer *et al.*, 2009). TGA1 and TGA4 (group I) control basal resistance independent of NPR1, despite their physical interaction (Shearer *et al.*, 2012); TGA2, TGA5, and TGA6 (group II) play crucial but redundant roles in systemic acquired resistance (Y. Zhang *et al.*, 2003; Kesarwani *et al.*, 2007); and TGA3 and TGA7 (group III) are involved in basal resistance and the regulation of *PR1* gene expression (Kesarwani *et al.*, 2007; Chen *et al.*, 2019). *BnGH17*, a member of the PR-2 protein group, encodes β-1,3-endoglucanase (Doxey *et al.*, 2007). Our results suggested a role for *BnTGA7* in activating *BnGH17* expression in response to *S. sclerotiorum* infection in *B. napus*. These findings advanced our understanding of the TGA family in regulating *PR-2* gene expression in the defense response. It was recently shown that overexpression of *BnNPR1* enhanced resistance to *S. sclerotiorum* in *B. napus* (Wang *et al.*, 2020a), suggesting that *BnNPR1* plays a positive regulatory role in defense responses to *S. sclerotiorum* infection. Whether activation of *BnGH17* by BnTGA7 depends on NPR1-mediated enhancement of DNA binding activity remains to be determined.

**Fig. 6.**
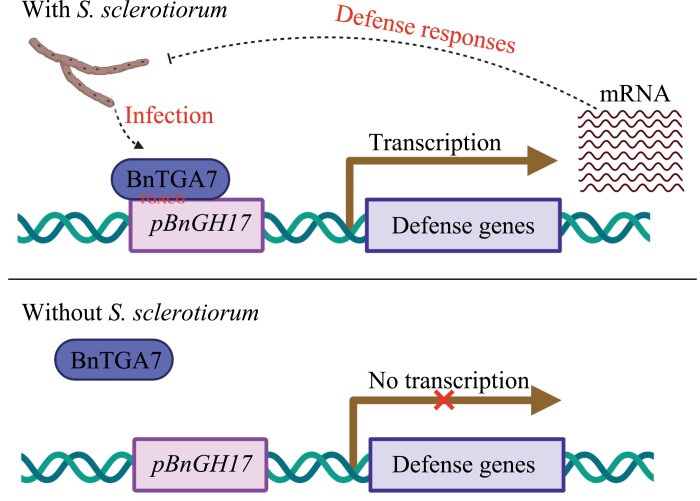
A proposed model illustrating that the *BnGH17* promoter (*pBnGH17*) activity was regulated by BnTGA7 upon *S. sclerotiorum* infection. BnTGA7 directly binds to the TGACG motif of *pBnGH17* and activates expression of *BnGH17* exclusively under *S. sclerotiorum* infection. This figure was generated with BioRender (http://biorender.com/).

In summary, we established a new strategy for enhancing *Sclerotinia* resistance with minimal adverse effects in *B. napus*. The *S. sclerotiorum*-inducible promoter *pBnGH17*, which harbors the TGACG-motif between positions –1432 and –1427, is essential for the *S. sclerotiorum* response (Fig. 6). *BnTGA7* directly binds to the TGACG motif in *pBnGH17* and activates transcriptional expression of *BnGH17* (Fig. 6). Furthermore, *pBnGH17*^*D7*^ was successfully utilized to drive SSR resistance in *B. napus* via the HIGS technique. As *S. sclerotiorum* is a typical necrotrophic fungal pathogen with a wide range of hosts, this work will also offer reference for engineering SSR resistance into other important economic crops such as soybean, sunflower, and peanut.

## Supplementary data

The following supplementary data are available at *JXB* online.

Fig. S1. Tissue-specific expression pattern of BnaC01g04530D, BnaA05g24230D, BnaC01g36130D, and BnaCnng78710D.

Fig. S2. The expression patterns of *BnGH17* under various stress conditions.

Fig. S3. GUS expression analysis in *pBnGH17*:*GUS* transgenic *A. thaliana* plants.

Fig. S4. Expression analysis of *BnTGA7* and *BnGH17* in leaves of *B. napus* after inoculation with *S. sclerotiorum*.

Fig. S5. Length distribution of target gene-specific siRNAs.

Fig. S6. The expression levels of *SsPG1* in *S. sclerotiorum*-infected *B. napus* cotyledons and leaves.

Fig. S7. Assessment of the disease resistance of HIGS transgenic *B. napus* (T_2_) to *S. sclerotiorum* by the stem inoculation method in the field.

Table S1. Primers used for qRT-PCR, gene cloning, and vector construction in this study.

Table S2. Agronomic traits of HIGS transgenic lines in the T_2_ generation.

erac328_suppl_Supplementary_Material

## Data Availability

All data supporting the findings of this study are available within the paper and within its supplementary data published online.
