# Supplementary material for: The Sclerotinia sclerotiorum-inducible promoter pBnGH17D7 in Brassica napus: isolation, characterization, and application in host-induced gene silencing
Source: J Exp Bot. 2022 Aug 5;73(19):6663–77. doi: 10.1093/jxb/erac328 (PMC9629790; doi:10.1093/jxb/erac328)
Supplement: erac328_suppl_Supplementary_Material [file erac328_suppl_supplementary_material.pdf]

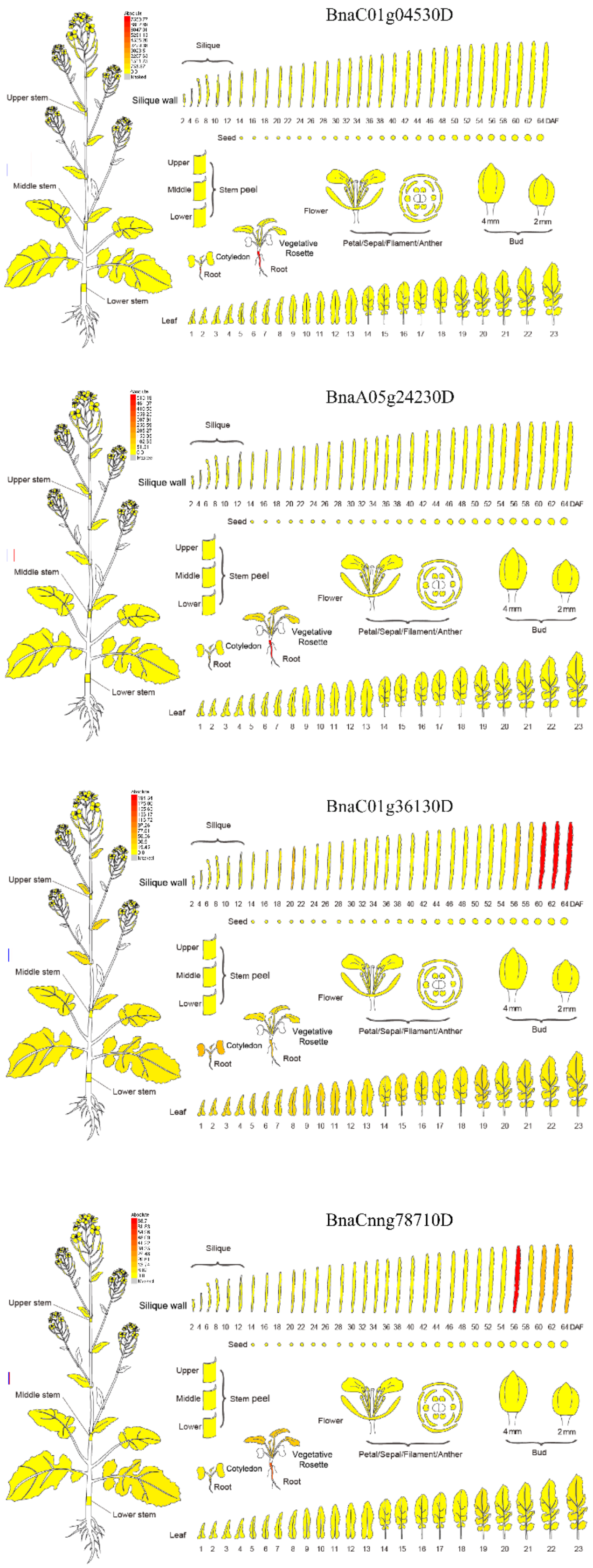

Figure S1. Tissue-specific expression pattern of BnaC01g04530D, BnaA05g24230D, BnaC01g36130D and BnaCnng78710D.

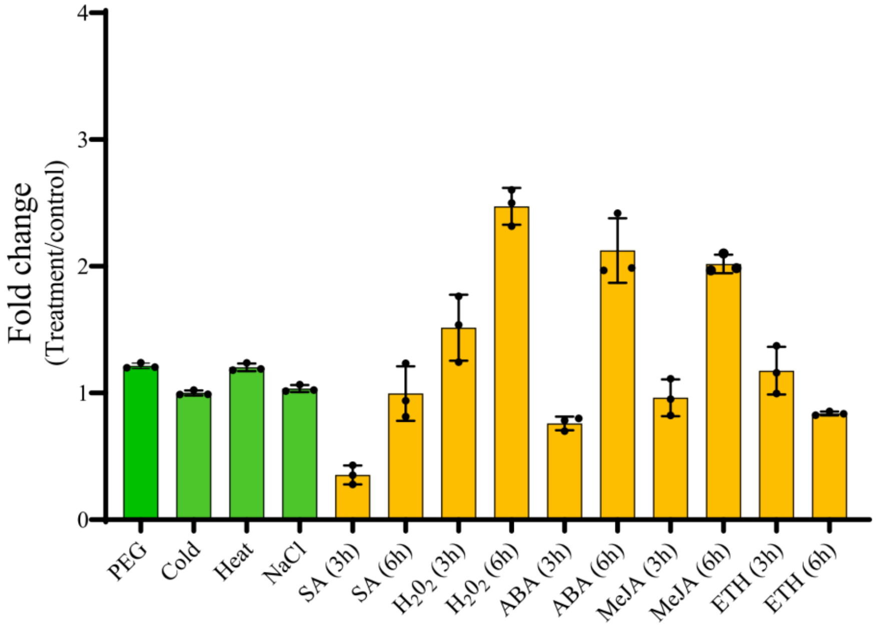

Figure S2. The expression patterns of *BnGH17* under various stress conditions. qRT-PCR analysis revealed the expression patterns of *BnGH17* under abiotic stress (PEG, cold, heat and NaCl treatments; green bar); hormone treatments (salicylic acid (SA), abscisic acid (ABA), methyl jasmonate (MeJA) and ethephon (ETH)) and H_2O_2 treatment (orange bar). *BnUBC10* (BnaA10g06670D) was used as reference gene. The values are presented as the mean ± SD of three independent biological replicates. Statistical significant differences between the control and each treatment was determined by Student's *t* test.

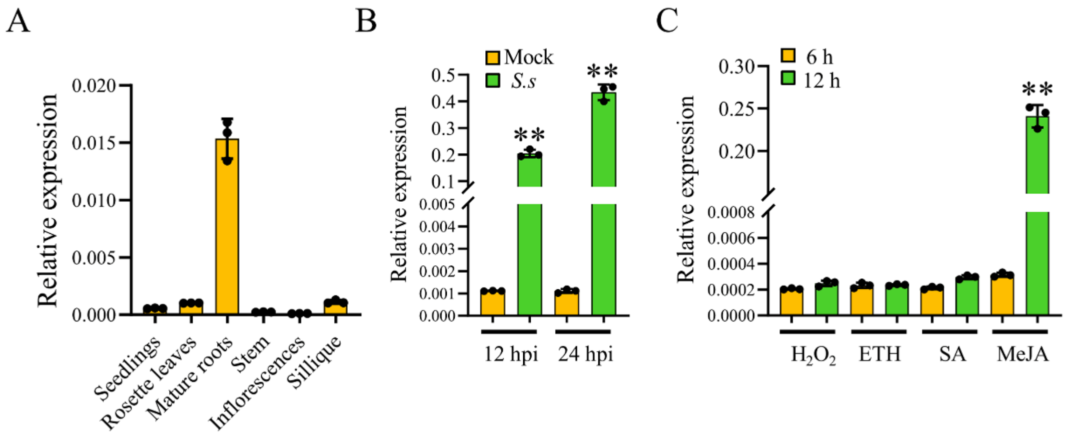

Figure S3. GUS expression analysis in *pBnGH17:GUS* transgenic *A. thaliana* plants. qRT-PCR analysis of *GUS* expression in diverse tissues (A), in leaves after *S.*

sclerotiorum inoculation (B) and in leaves after H_2O_2, SA, MeJA and ETH treatments (C). *AtUBQ10* (At5g53300) was used as reference gene. The values are presented as the mean ± SD of three independent biological replicates. The asterisks indicate significant differences (***p* < 0.01, Student's *t* test).

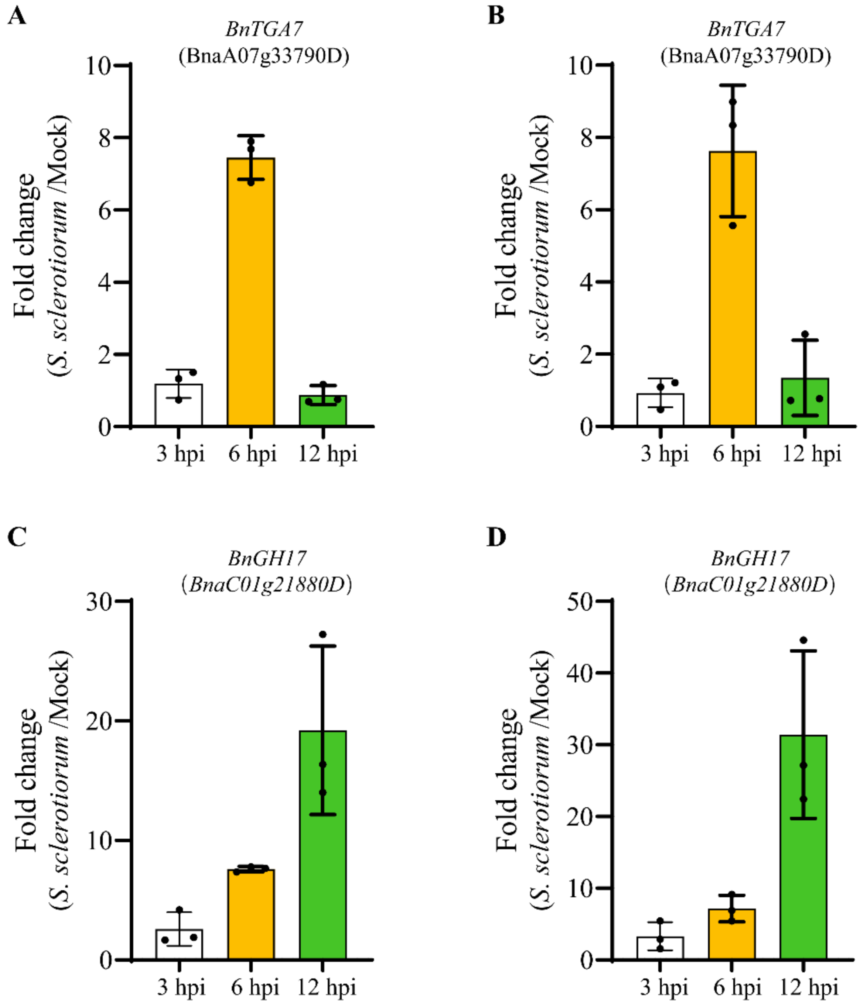

Figure S4. Expression analysis of *BnTGA7 and BnGH17* in leaves of *B. napus* after inoculation with *S. sclerotiorum*. *BnUBC9* (A, C) and *BnUBC10* (B, D) were used as reference genes, respectively. The values are presented as the mean ± SD of three independent biological replicates. hpi, hours post-inoculation.

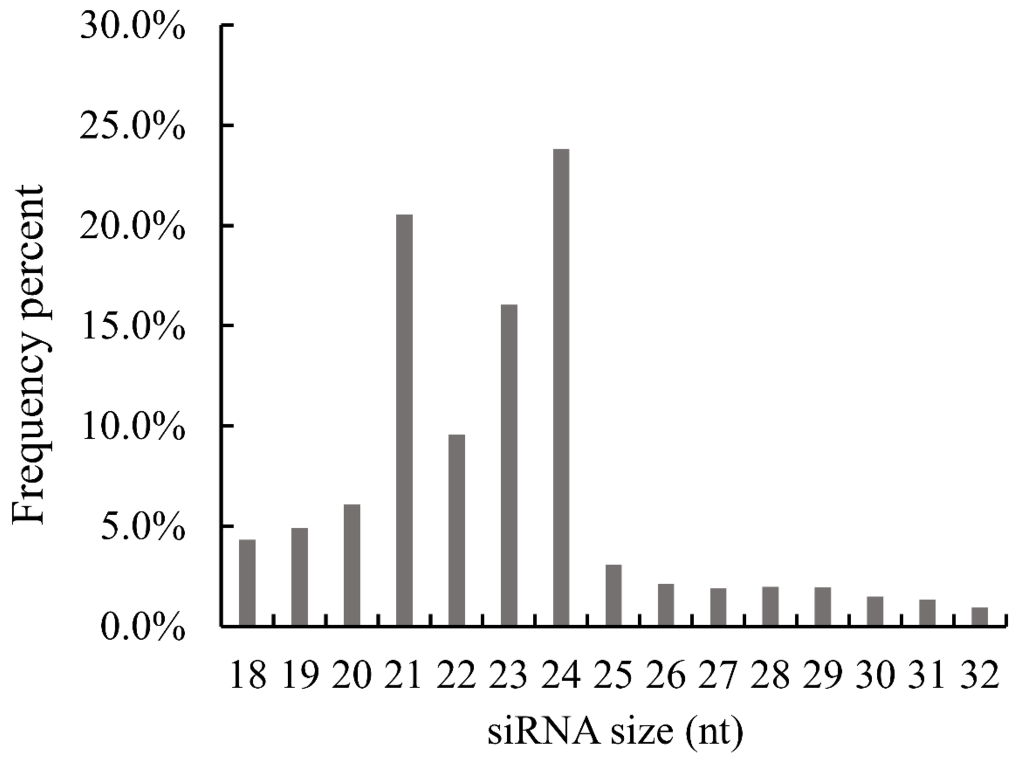

Figure S5. Length distribution of target gene-specific siRNAs.

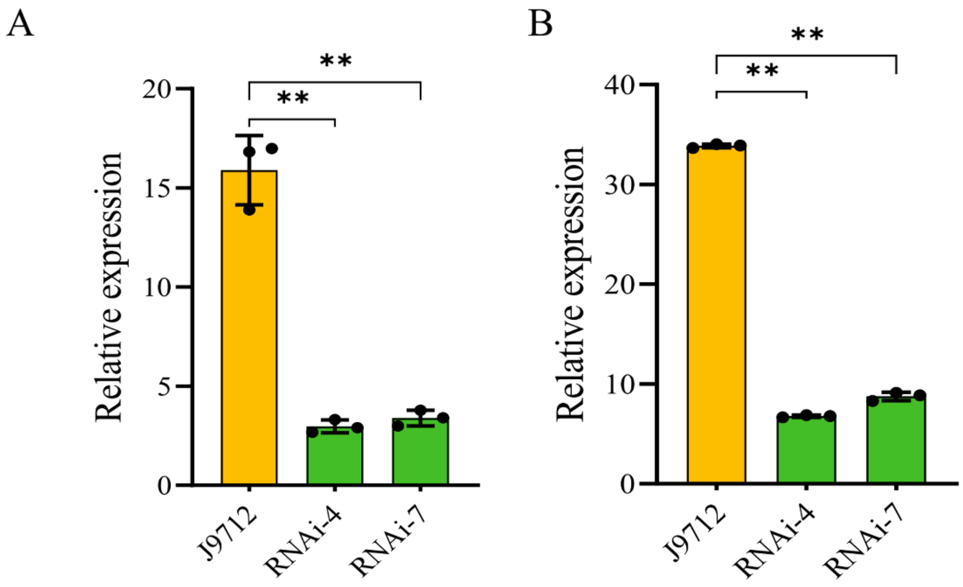

Figure S6. The expression levels of *SsPG1* in *S. sclerotiorum*-infected *B. napus* cotyledons (A) and leaves (B). Values were normalized to the fungal endogenous control gene *SsActin* (SS1G_08733). The values are presented as the mean ± SD of

three independent biological replicates. The asterisks indicate significant differences (**$p < 0.01$, Student's t test).

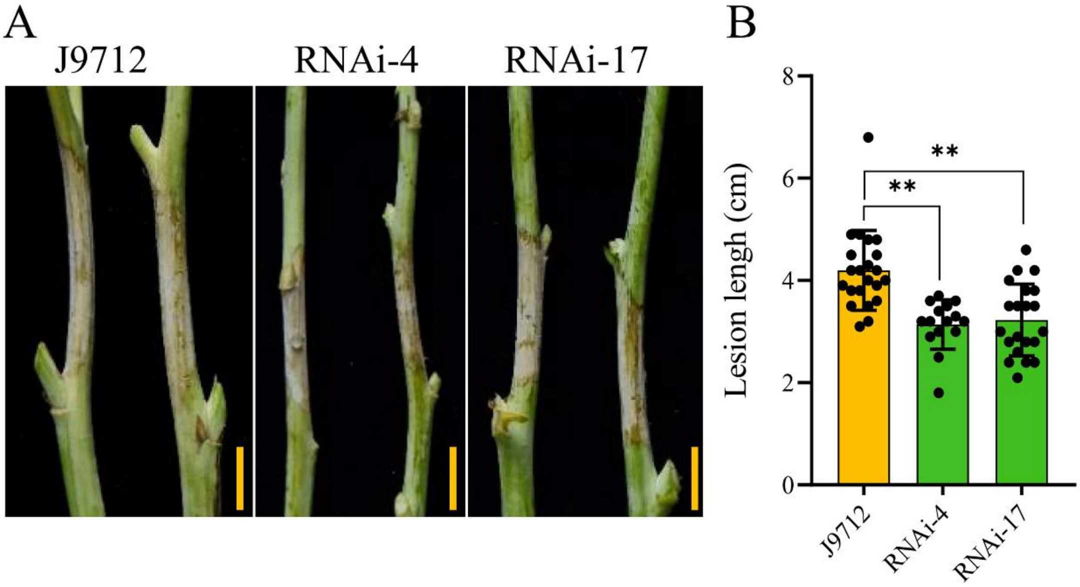

Figure S7. Assessment of the disease resistance of HIGS transgenic *B. napus* (T_2) to *S. sclerotiorum* by the stem inoculation method in the field. (A) Symptoms of *S. sclerotiorum* infection on *B. napus* stems at 7 d post-inoculation (dpi). Scale bars= 1 cm. (B) Quantification of the lengths of lesions on stems at 7 dpi.

Table S1. Primers used for qRT-PCR, gene cloning and vector construction in this study.

Primer name	Primer sequence (5'-3')	Description
BnGH17-F	GAAACGCTGCGACTTTTGATAA	qPCR for *BnGH17* in *B. napus*
BnGH17-R	CATCGAACATGGCGAACAAATA	
BnUBC10-F	GAAGACATGTTTCATTGGCAGGC	qPCR for *BnUBC10*, reference gene of *B. napus*
BnUBC10-R	CTTAGGAGGTTTGAAAGGGTAATCA	
BnUBC9-F	TCCATCCGACAGCCCTTACTCT	qPCR for *BnUBC9*, reference gene of *B. napus*
BnUBC9-R	ACACTTTGGTCCTAAAAGCCACC	
qBnTGA7-F	TCGGGCATGTGGAGAACTTG	qPCR for *BnTGA7* (BnaA07g33790D) in *B. napus*
qBnTGA7-R	AAGATTCTCTAACGCTGCG	
AtEF-1α-F	TGAGCACGCTCTTCTTGCTTTCA	qPCR for *AtEF-1α*, reference gene of *Arabidopsis*
AtEF-1α-R	GGTGGTGGCATCCATCTTGTTACA	
AtUBQ10F	GACGCTTCATCTCGTCC	qPCR for *AtUBQ10*, reference gene of *Arabidopsis*
AtUBQ10R	GTAAACGTAGGTGAGTCCA	
GUS-F	AGTGAAGGGCGAACAGTTCCTGAT	qPCR for *GUS* in *Arabidopsis*
GUS-R	TTCAGCGTAAGGGTAATGCGAGGT	
SsTub1qF	ACCTCCATCCAAGAACTC	qRT-PCR for *Sstub1*, reference gene of *S. sclerotiorum*
SsTub1qR	GAACTCCATCTCGTCCAT	
SsActinqF	CCCCAGCGTTCTACGTCT	qRT-PCR for *SsActin*, reference gene of *S. sclerotiorum*
SsActinqR	CATGTCAACACGAGCAATG	
WJP185F	CAAGATGACTGTCTCGCTAT	qRT-PCR for *SsPG1* in S. sclerotiorum
WJP185R	GGTGGAGGATTCAATGATAAC	
pBnGH17-F	CCAAAGGAAGACAAGGAGCA	Gene cloning for *pBnGH17*
pBnGH17-R	AGCAAGAAGAAGATACAGTGGGA	
D1-F	CAGGTCGACTCTAGAGGATCCTCTTGTCTATGAATACGTGG	Gene cloning for D1
D1-R	GGACTGACCACCCGGGGATCCATCGAAATTGATGTG	
D2-F	CAGGTCGACTCTAGAGGATCCGGACCACCACAAAGGTCA	Gene cloning for D2
D2-R	GGACTGACCACCCGGGGATCCATCGAAATTGATGTG	
D3-F	CAGGTCGACTCTAGAGGATCCTGTTTCTCGTCATTCGTT	Gene cloning for D3
D3-R	GGACTGACCACCCGGGGATCCATCGAAATTGATGTG	
D4-F1	CCAAAGGAAGACAAGGAGCA	Gene cloning for D4
D4-R1	AACAACGAGTTTTCTGAACG	
D4-F2	TCGTTGTTTCTCGTCATTCG	
D4-R2	AGCAAGAAGAAGATACAGTGGGA	
D5-F1	CAGGTCGACTCTAGAGGATCCACGTG ATTGATTTTC	Gene cloning for D5
D5-R1	AACAACGAGTTTTCTGAACG	
D5-F2	TCGTTGTTTCTCGTCATTCG	
D5-R2	AGCAAGAAGAAGATACAGTGGGA	
D6-F1	CCAAAGGAAGACAAGGAGCA	Gene cloning for D6
D6-R1	AACACGTCATATAGTTCCAA	
D6-F2	GACGTGTTTCTCGTCATTCG	

D6-R2	AGCAAGAAGAAGATACAGTGGGA	
D7-F1	CCAAAGGAAGACAAGGAGCA	Gene cloning for D7
D7-R1	AACACTCAAAAGCACATTAG	
D7-F2	TGAGTGTTTCTCGTCA TTCG	
D7-R2	AGCAAGAAGAAGATACAGTGGGA	
D7-mut-F1	TGCCTGCAGGTCGACTCTAGACTTTGTAAACGAAGC	Gene cloning for D7-mut
D7-mut-R1	CCCTTATAGTTCCAAGCG	
D7-mut-F2	CGCTTGGAACTATAAGGG	
D7-mut-R2	GGACTGACCACCCGGGGATCCCACCCCTTATAGTTC	
pBnGH17-F1	CGCTCTAGAACTAGTGGATCCCACGTCATATAGTTC	Vector construction for *pBnGH17*^{D7}::luciferase
pBnGH17-R1	CGCTCTAGAACTAGTGGATCCCACGTCATATAGTTC	
pBnGH17-F2	GACTCACTATAGGGCGAATTCCCAAAGGAAGACAAG	Vector construction for pHIS2-*pBnGH17*^{D7}
pBnGH17-R2	TCACGTCATATAGTTGATTCGCGAACGCGTGAGCTC	
pBnGH17-F3	ATTCCCGGGGAGCTCACGCGTTTTTGAGACAGAGCT	Vector construction for pHISi-1-*pBnGH17*^{D7}
pBnGH17-R3	ATAATGCCAGGAATTTCTAGACGTCATATAGTTCCA	
BnTGA3-F	TTAAGTGCGTTCCCGTG	Gene cloning for *BnTGA3*
BnTGA3-R	CTAGATGATGAGCTCTTC	
BnTGA7-F	CGATGCTTCATTCTGAC	Gene cloning for *BnTGA7*
BnTGA7-R	TTAAGTTGGTTCTCGTGG	
BnTGA7-F1	CGCTCTAGAACTAGTGGATCCATGCTTCATTCTGTT	Vector construction for 35s::BnTGA7
BnTGA7-R1	GATAAGCTTGATATCGAATTCTTAAGTTGGTTCTCG	
BnTGA7-F2	TTCCAGGGGCCCCTGGGATCCATGCTTCATTCTGTT	Vector construction for 35s::BnTGA7-GST
BnTGA7-R2	CTCGAGTCGACCCGGGAATTCTTAAGTTGGTTCTCG	
BnTGA3-F1	CGCTCTAGAACTAGTGGATCCATGATGAGCTCTTCT	Vector construction for 35s::BnTGA3
BnTGA3-R1	GATAAGCTTGATATCGAATTCTTAAGTGCGTTCCCG	
pBnGH17-F4	GTTTAAACTATCAGTGTTTGACACGTCATATAGTTC	pBnGH17^{D7} was cloned into pMDC83-ihpRNAi
pBnGH17-R4	ACTAGTTAATTAAGAATTAGCAGCAAGAAGAAGATA	
SsPG1-F	GGAGAGGACCTCGACTCTAGA	Identification of positive transgene for HIGS transgenic plants
SsPG1-R	GGATCCCGAGACAGTCATCTTG	

Table S2. Agronomic traits of HIGS transgenic lines in the T_2 generation.

Line	Plant height（cm）	First effective branch number	Silique number per plant	Silique lengh (cm)	Silique seed number	Thousand-seed weight（g）	Yield per plant (g)	Oil content（%）	C18:1（%）
J9712	152.0 ± 6.0	8.2 ± 0.5	227.9 ± 16.9	8.3 ± 0.2	25.6 ± 0.8	3.0 ± 0.0	22.8 ± 3.7	42.7 ± 1.2	66.0 ± 2.2
RNAi-4	152.2 ± 0.9	7.4 ± 0.2	264.1 ± 39.6	8.1 ± 0.2	25.7 ± 0.8	3.1 ± 0.1	21.1 ± 3.9	42.2 ± 1.7	65.5 ± 2.3
RNAi-7	152.1 ± 5.9	8.4 ± 1.2	232.6 ± 11.7	8.6 ± 0.7	25.1 ± 0.7	3.3 ± 0.1	26.2 ± 5.1	43.2 ± 1.6	65.8 ± 2.4
